# Treatment-resistant nephrotic syndrome in dense deposit disease: complement-mediated glomerular capillary wall injury?

**DOI:** 10.1007/s00467-020-04600-9

**Published:** 2020-05-23

**Authors:** Caroline Duineveld, Eric J. Steenbergen, Andrew S. Bomback, Nicole C. A. J. van de Kar, Jack F. M. Wetzels

**Affiliations:** 1 Department of Nephrology, Radboud University Medical Center, PO BOX 9101, 6500 HB Nijmegen, Netherlands; 2 Department of Pathology, Radboud University Medical Center, Nijmegen, Netherlands; 3 Department of Medicine, Division of Nephrology, Columbia University Medical Center, New York, NY USA; 4 Department of Pediatric Nephrology, Radboud Institute for Molecular Life Sciences, Amalia Children’s Hospital, Radboud University Medical Center, Nijmegen, Netherlands

**Keywords:** C3 glomerulopathy, Dense deposit disease, Podocyte, Nephrotic syndrome

## Abstract

**Background:**

The C3 glomerulopathies (C3G) are recently defined glomerular diseases, attributed to abnormal complement regulation. Dense deposit disease (DDD) is part of the spectrum of C3G, characterized by electron-dense deposits in the lamina densa of the glomerular basement membrane. Patients with DDD present with hematuria, variable degrees of proteinuria, and kidney dysfunction. Kidney biopsies typically disclose proliferative and inflammatory patterns of injury. Treatment with glucocorticoids and mycophenolate mofetil has been shown to achieve remission of proteinuria in a significant proportion of C3G patients.

**Case-diagnosis/treatment:**

We report two patients with persistent nephrotic syndrome while on immunosuppressive therapy. Repeat kidney biopsies disclosed massive C3 deposits with foot process effacement in the absence of proliferative or inflammatory lesions on light microscopy.

**Conclusion:**

These cases, coupled with data from animal models of disease and the variable response to eculizumab in C3G patients, illustrate that two different pathways might be involved in the development of kidney injury in C3G: a C5-independent pathway leading to glomerular capillary wall injury and the development of proteinuria versus a C5-dependent pathway that causes proliferative glomerulonephritis and kidney dysfunction.

**Electronic supplementary material:**

The online version of this article (10.1007/s00467-020-04600-9) contains supplementary material, which is available to authorized users.

## Introduction

Dense deposit disease (DDD) is a rare renal disease, within the spectrum of the C3 glomerulopathies (C3G), caused by complement dysregulation [1]. DDD is characterized by electron-dense deposits in the lamina densa with a ribbon or a sausage structure. Patients with DDD present with hematuria, proteinuria, and/or kidney dysfunction. The initial kidney biopsy mostly discloses a mesangial or a membranoproliferative glomerulonephritis (MPGN), but also endocapillary proliferation and crescents can be observed [1]. Treatment with glucocorticoids and mycophenolate mofetil (MMF) benefits a significant proportion of patients [2]. Although complement dysregulation is pivotal in the pathogenesis of C3G, the causative role of the various complement proteins is debated. Studies in mouse models suggest an important role for the C5a fragment and its receptor (C5aR1) [3]. Treatment with C5aR blockade is now under investigation in C3G (NCT03301467). We report two patients with DDD with improved kidney function but persistent proteinuria after immunosuppressive therapy. A kidney biopsy showed evidence of podocyte injury in the absence of proliferative lesions. These cases illustrate how two different, complement-dependent pathways might cause kidney injury in DDD.

## Case reports

### Case 1

This Caucasian female patient presented in 2008, at the age of 17 years, with nephrotic syndrome (urine protein-creatinine ratio (UPCR) of 9 g/10 mmol (normal < 0.10 g/10 mmol), serum albumin 19 g/L (normal > 35 g/L)). Serum creatinine concentration was normal (85, normal 45–90 μmol/L). The C3 concentration was low (196, normal 700–1500 mg/L) with a normal C4 concentration (216, normal 100–400 mg/L). Anti-nuclear antibodies were negative. The kidney biopsy (D+2) (Supplementary Fig. S1A-C) showed a diffuse endocapillary and mesangial proliferative glomerulonephritis with extracapillary proliferation in 70% of the glomeruli. Ribbon-like eosinophilic deposits were seen in the capillary walls. There was C3-dominant staining on immunofluorescence (IF) (Supplementary Fig. S1B), and electron microscopy (EM) was typical for DDD (Supplementary Fig. S1C).

No genetic variants in complement regulatory genes were detected. No auto-antibodies against factor H nor C3 nephritic factor (C3NeF) activity were found. The patient was treated (D+3) with methylprednisolone pulses (3 × 1000 mg i.v.), followed by oral prednisolone (1 mg/kg), MMF (2 × 1000 mg), and conservative therapy with an ACE inhibitor. Despite treatment, kidney function deteriorated (serum creatinine rising from 85 to 283 μmol/L), necessitating introduction (D+16) of cyclophosphamide and plasmapheresis (total of 10 sessions) with temporary interruption of MMF. After 6 weeks, cyclophosphamide was discontinued (D+56), and MMF restarted. Kidney function improved, with serum creatinine reaching a nadir of 73 μmol/L (MDRD 89 ml/min/1.73m^2^) after 1 year; however, proteinuria persisted in nephrotic range (UPCR 3.5–4.5 g/10 mmol, serum albumin 33 g/L) (Supplementary Fig. S2). The dose of MMF was initially tapered. In 2012, because of increasing proteinuria (UPCR 7.3 g/10 mmol) and decreasing serum albumin (28 g/L), the patient was treated with methylprednisolone pulses, and the MMF dose was again increased to 2 × 1000 mg. Despite treatment, proteinuria persisted (UPCR 4–5 g/10 mmol, serum albumin 22–27 g/L). Over the next years, after a *Salmonella* infection in 2013, the patient developed progressive gastro-intestinal intolerance to MMF. In 2017 the drug was stopped, and the side effects disappeared. Because of low blood pressure, the ACE inhibitor was also discontinued. In view of the persistent nephrotic syndrome, with a slow decline in kidney function (serum creatinine 92 μmol/L, MDRD 63 ml/min/1.73m^2^), treatment with C5aR blockade was discussed, and a kidney biopsy was performed. The kidney biopsy, taken 10 years after initial diagnosis, showed a sclerosing glomerulopathy (50% globally sclerosed glomeruli) with massive deposits of eosinophilic material in the mesangium and capillary walls. There was no cellular proliferation nor active inflammation. Complete foot process effacement was seen on electron microscopy (Supplementary Fig. S1D-F). In the absence of inflammation, ongoing C3 deposition was deemed responsible for the proteinuria. A superimposed minimal change nephropathy (MCD), analogous to the nephrotic syndrome in class I or II lupus nephritis (i.e., lupus podocytopathy) or IgA nephropathy, was considered a possibility. These lesions typically respond to steroid or calcineurin inhibitor therapy akin to primary podocytopathies. In light of this, our patient received three pulses of methylprednisolone, followed by prednisolone (1 mg/kg) and tacrolimus without any effect on the level of proteinuria or serum albumin. At the most recent follow-up visit (11 years after diagnosis), she remains nephrotic (UPCR 4.2 g/10 mmol, serum albumin 28 g/L), with a serum creatinine of 104 μmol/L (MDRD 54 ml/min/1.73m^2^). Blood pressure was well controlled (111/67 mmHg).

### Case 2

This Caucasian female patient presented in 2004, at the age of 6 years, with nephrotic syndrome (urine protein level 19.8 g/L, serum albumin 21 g/L). There was mild kidney dysfunction (serum creatinine 61 μmol/L). C3 concentration was low (84 mg/L), with normal C4 concentration (108 mg/L). A kidney biopsy showed a membranoproliferative glomerulonephritis with mild endocapillary proliferation and, in few glomeruli, extracapillary proliferation. Ribbon-like eosinophilic deposits were seen in the capillary walls. Dominant C3 deposits were observed, and EM disclosed the typical appearance of DDD (Supplementary Fig. S3A-C).

No genetic variants were found in complement regulatory genes. C3NeF was negative, and no auto-antibodies against factor H were detected. She was treated with high dose prednisolone and conservative therapy with an ACE inhibitor. She reached complete remission, and prednisolone was discontinued after 5 years. At the age of 15 years, she developed recurrent nephrotic syndrome (serum creatinine 87 μmol/L, serum albumin 21 g/L, UPCR 6.4 g/10 mmol) (Supplementary Fig. S4). No obvious trigger was found. Treatment consisted of methylprednisolone pulses, followed by oral prednisolone and mycophenolic acid (MPA), with improvement of kidney function (serum creatinine 63 μmol/L, MDRD > 90 ml/min/1.73m^2^) and complete remission of proteinuria. The prednisolone was stopped. At the age of 18 years, she again presented with a recurrence. Laboratory results showed a serum creatinine of 64 of μmol/L, a serum albumin of 26 g/L, and a UPCR of 3.1 g/10 mmol. Despite an increase in dose of MPA, the nephrotic syndrome persisted, and, at the age of 21 years, a kidney biopsy was performed to evaluate eligibility for anti-complement therapy. The biopsy showed a sclerosing glomerulopathy with massive eosinophilic deposits in the mesangium and capillary walls. The typical hyperlobular MGPN pattern was not observed. No cellular proliferation nor active inflammation was present. There was extensive foot process effacement (Supplementary Fig. S3D-F). At the most recent follow-up visit (15 years after diagnosis), proteinuria was 2.7 g/10 mmol (with a low serum albumin 24 g/L), with a preserved kidney function (serum creatinine of 64 μmol/L, MDRD >90 ml/min/1.73m^2^) and a normal C3 concentration (1010 mg/L). Blood pressure is well controlled (120/78 mmHg) with the use of an angiotensin II receptor blocker.

## Discussion

**Fig. 1:**
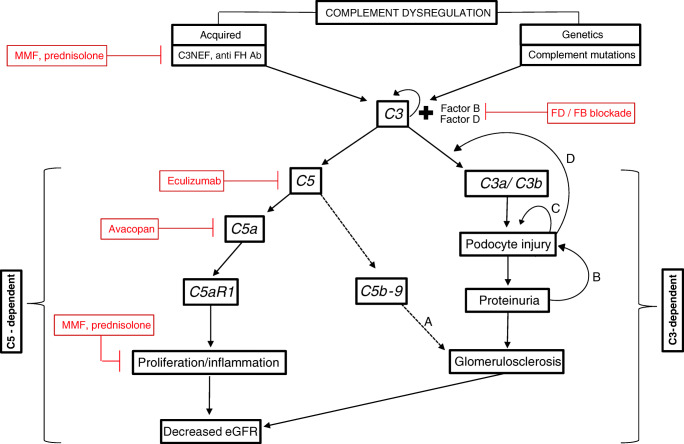
Pathways of kidney injury in C3 glomerulopathies

C3G is attributed to complement dysregulation. Recent reviews describe the two pathways that may be involved in kidney injury [1, 4]. We suggest that our cases illustrate that two pathways exist, with differential types of glomerular injury (Fig. 1). One pathway is C5 independent and causes injury to the glomerular capillary wall, characterized by proteinuria and podocyte foot process effacement, while the other pathway is C5 dependent and responsible for proliferative glomerulonephritis and kidney dysfunction. We hypothesize that in our patients, the C5-dependent route was managed effectively with MMF and corticosteroids, but the C5-independent injury persisted. In contrast to podocyte injury that is associated with primary GNs (e.g., lupus podocytopathy, IgAN with superimposed MCD), our patients did not respond to steroids or calcineurin inhibitor therapy, suggesting a complement rather than circulating factor-induced mechanism of injury.

Studies in mouse models nicely recapitulate this sequence of events. Pickering et al. created a factor H-deficient mouse model and showed that these mice developed MPGN, characterized by glomerular proliferation, and formation of crescents [3]. C5 deficiency, studied by crossing factor H deficient with C5-deficient mouse, resulted in markedly reduced proliferative and inflammatory lesions and improvement of eGFR. Subsequent studies showed that the development of mesangial and glomerular capillary hypercellularity was prevented in C5-deficient mice, but not in mice deficient for C6 or C9 [5], clearly underlining the role of C5a/C5aR in the development of proliferative C3G. However, C5 deficiency did not fully reverse the disease. In fact, the factor H- and C5-deficient mice still developed GBM thickening and subendothelial deposits, and proteinuria was not attenuated. Together, these findings support a role for C3, independent of C5 activation, in the development of proteinuria [3]. The anaphylatoxin C3a and its receptor C3aR may be involved in kidney injury. Mice injected with Stx2/LPS develop a HUS phenotype (thrombocytopenia, glomerular capillary thrombosis, and acute kidney injury) with excessive glomerular C3 deposits and injury and loss of podocytes. Podocyte injury and loss could be prevented by treatment with a C3aR antagonist. Blockade of the C3aR also reduced proteinuria [6]. In other experimental models, blockade of the C3a receptor also was effective in reducing proteinuria and glomerulosclerosis [7, 8], and several possible mechanisms of C3a-induced glomerular injury have been suggested [6, 9]. Alternatively, C3dg deposition could be involved, akin to the different pathways of complement-mediated injury in paroxysmal nocturnal hemoglobinuria (PNH). In PNH a C5b-9-dependent pathway leads to membrane-attack-complex-mediated intravascular hemolysis, and a C3-dependent pathway results in deposition of the opsonin C3dg on the erythrocyte, explaining ongoing extravascular hemolysis in patients treated with eculizumab [10]. Accordingly, C3 may play a role, either through C3aR activation or C3dg deposition, in inducing glomerular capillary wall injury, leading to proteinuria, podocyte loss, and glomerulosclerosis in C3G.

A detailed evaluation of the response to treatment with eculizumab, which inhibits C5 activation, provides further support for the C5-independent kidney injury. In a recent study of 4 patients with C3GN and 6 patients with immune complex MPGN, treatment with eculizumab had no significant effect on proteinuria in 7 patients, despite normalization of the elevated sC5b-9 levels in all patients [11]. Le Quintrec et al. reported 26 patients with C3G who were treated with eculizumab. A very good response was noted in only six patients. These patients were characterized by a rapidly progressive glomerulonephritis and the presence of crescents in the kidney biopsy [12]. Supplementary Table S1 summarizes case reports with information of kidney biopsies performed to evaluate the histological response to eculizumab. A decrease in proliferation was noted in 15/23 cases (65%). In contrast, a significant decrease in C3 staining was observed in only 2 patients. Clinical characteristics at start of therapy were quite variable. However, there was a clear tendency for improvement in eGFR, whereas proteinuria response was more variable. Of note, nephrotic range proteinuria and/or nephrotic syndrome persisted in 6 patients, despite a decrease in proliferation.

Our patients are not unique. Figueres et al. reported a patient with DDD (case 2), who presented with nephrotic syndrome and had repeated kidney biopsies during follow-up. The first kidney biopsy, performed because of persistent nephrotic syndrome, showed moderate mesangial proliferation and no inflammatory cell influx. A second biopsy, performed while on steroid therapy with persistent non-nephrotic proteinuria, showed no mesangial proliferation but evident mesangial and subendothelial deposits characteristic of DDD. A third biopsy 20 years after disease onset, performed because of deteriorating kidney function and nephrotic range proteinuria, showed severe global glomerulosclerosis (58%) and interstitial fibrosis and tubular atrophy (60%) [13].

In summary, we report two DDD patients with persistent nephrotic syndrome despite immunosuppressive therapy, with no inflammatory lesions in the kidney biopsy. We propose that C3 deposition causes glomerular capillary wall injury leading to proteinuria and podocyte loss, independent of C5, explaining inefficacy of current treatment modalities such as eculizumab, and suggesting potential efficacy of novel complement inhibitors working at the level of C3. We would like to emphasize that C3G is a heterogeneous disease and that response to therapy is also quite variable. Our patients illustrate one part of the disease spectrum. There is a need for large clinical registries and clinical trials with detailed phenotyping of patients and evaluation of treatment response.

We propose that two pathways are involved in the development of kidney injury in C3G. The first pathway is a C5-dependent pathway. Degradation of C5 leads to the production of the fragments C5a and C5b, the latter being assembled into the membrane attack complex: C5b-9. C5a is a strong proinflammatory mediator which binds to the C5a receptor 1 (C5aR1). C5aR1 is expressed on immune cells and various cells types in the kidney such as mesangial cells, glomerular endothelial cells, and epithelial cells, and expression may be up-regulated in case of tissue injury. Glomerular inflammation may lead to acute kidney injury and can be responsive to treatment with eculizumab, a C5a receptor blocker, and/or standard immunosuppressants. The generation of the membrane attack complex may augment glomerulosclerosis and tubulo-interstitial fibrosis (arrow A), but not proteinuria, as demonstrated by Turnberg et al. in mice deficient of a MAC formation regulator [14].

The second pathway is C3 dependent but C5 independent. In this pathway degradation of C3 leads to the following: (1) the production of C3b, which attaches on the endothelial cell surface and GBM, and is further cleaved into C3dg, and (2) the generation of the anaphylatoxin C3a, which can bind to the C3a receptor, which is present on endothelial and epithelial cells. The role of the alternative complement pathway in proteinuric kidney diseases has been studied using animal models, including a model of toxic podocyte injury [14] and a model with protein overload [9]. In these models initial podocyte injury (either induced by Adriamycin or protein overload) resulted in glomerular C3 deposition, podocyte injury and loss, proteinuria, glomerular sclerosis, tubulo-interstitial fibrosis and acute kidney injury. The kidney injury could be attenuated by inducing complement deficiencies (C3, factor D or factor B deficiency) and could be increased in mice deficient in complement regulators (factor H deficiency) [9, 14]. Although it is evident from these studies that the alternative complement pathway plays a role in the development of podocyte injury, proteinuria, and glomerulosclerosis, it is uncertain how C3 induces kidney injury. We propose that complement deposition induces glomerular capillary wall injury leading to proteinuria and podocyte loss. Both C3a and C3b/C3dg may contribute to this process. Podocyte injury may lead to further podocyte loss, as damaged podocytes can injury adjacent podocytes [15]. Thus, proteinuria and podocyte injury may induce a vicious circle of injury with ongoing damage via complement-mediated and other, yet unknown, mechanisms (arrows B–D). Therapeutic interventions may be C3, factor B, or factor D blockade; selective blockade of the C3a receptor; and/or standard immunosuppressive therapy in patients with antibody-mediated C3G.

## Electronic supplementary material

ESM 1 (DOCX 51 kb)

Fig S1A (JPG 841 kb)

Fig S1B (JPG 565 kb)

Fig S1C (JPG 2617 kb)

Fig S1D (JPG 365 kb)

Fig S1E (JPG 662 kb)

Fig S1F (JPG 234 kb)

Fig S2 (PPTX 90 kb)

Fig S3A (JPG 472 kb)

Fig S3B (JPG 548 kb)

Fig S3C (JPG 2588 kb)

Fig S3D (JPG 721 kb)

Fig S3E (JPG 802 kb)

Fig S3F (JPG 1760 kb)

Fig S4 (PPTX 87 kb)
