# Supplementary material for: Treatment-resistant nephrotic syndrome in dense deposit disease: complement-mediated glomerular capillary wall injury?
Source: Pediatr Nephrol. 2020 May 23;35(9):1791–5. doi: 10.1007/s00467-020-04600-9 (PMC7384995; doi:10.1007/s00467-020-04600-9)
Supplement: Supplementary file 1 — (DOCX 51 kb) [file 467_2020_4600_MOESM1_ESM.docx]

**Supplementary Data File**

Index:

Supplemental Figure S1A-C: Kidney biopsy of patient 1 at diagnosis (A-C) and after 10 years of follow-up (D-F) (Images and legend)

Supplemental Figure S2: Clinical course of patient 1 after diagnosis of DDD (Graph and legend)

Supplemental Figure S3A-F: kidney biopsy of patient 2 at diagnosis (A-C) and at follow-up (D-F) (Images and legend)

Supplemental Figure S4: Clinical course of patient 2 after diagnosis of DDD (Graph and legend)

Supplemental Table S1: Clinical parameters and histology of repeated kidney biopsies in patients treated with eculizumab

References of Supplemental Table S1

**Supplementary Figure S1: Kidney biopsy of patient 1 at diagnosis (A-C) and after 10 years of follow-up (D-F).**

Legend: the first kidney biopsy contained 12 glomeruli per cross section. In light microscopy all glomeruli showed endocapillary and mild mesangial hypercellularity and in 70% of the glomeruli signs of recent extracapillary proliferation were found. Ribbon-like eosinophilic deposits were seen in the capillary walls. There was mild mesangial hypercellularity (Panel A, MS staining, magnification 40x). On IF intense C3 staining (+++) was seen in the capillary walls, mesangium and tubular basement membranes (panel B). Staining for IgG, IgA, C1q was negative; staining for IgM, Kappa and Lambda was positive (+). On EM massive intramembranous dense deposits were seen in the basement membranes with extensive podocyte foot effacement and mild microvillous transformation of the podocytes (panel C).

The second biopsy, obtained 10 years after diagnosis, contained 30 glomeruli per cross section. On light microscopy the glomeruli showed a sclerosing glomerulopathy (50% globally sclerosed glomeruli, with 30% IFTA) with massive eosinophilic deposits in the mesangium and capillary walls. Panel D shows the marked increase in eosinophilic deposits in biopsy 2 compared to biopsy 1 (MS staining, magnification 40x). There was no active inflammation (no endocapillary proliferation or necrotizing/extracapillary proliferative lesions). On IF a marked increase in C3 staining (+++) was observed (panel E), in the capillary walls, mesangium and tubular basement membranes, with a trace of C1q, and negative staining for IgG, IgM, IgA, Kappa and Lambda. On EM extensive podocyte foot process effacement was seen, with intramembranous, mesangial and tubular basement membrane depositions and mild microvillous transformation (panel F).

Abbreviations: EM = electron microscopy, IF = immunofluorescence, MS = methenamine silver stain.

**Supplemental Figure S2: Clinical course of patient 1 after diagnosis of DDD**

Legend: after presentation with nephrotic syndrome the patient was treated with methylprednisolone pulses (3x 1000 mg i.v.) (*triangle*), followed by oral prednisolone (1mg/kg) (*dark blue bar*) and MMF (2x 1000 mg) (*light blue bar*). Despite treatment, serum creatinine increased, and the patient was started on plasmapheresis (10 sessions) (*yellow stars*) and cyclophosphamide (*yellow bar*). MMF was interrupted. After six weeks cyclophosphamide was discontinued and MMF was restarted (2x 1000 mg). Prednisolone and MMF were slowly tapered (lowest dose was 500 mg MMF and 5 mg prednisolone per day). Four years after diagnosis proteinuria increased (UPCR 7.3 mg/10 mmol creatinine) and serum albumin decreased (28 g/L). The patient was treated with methylprednisolone (3x 1000 mg i.v.) (*triangle*) and the MMF dose was increased (2x 1000 mg). Due to gastro-intestinal intolerance MMF was discontinued 9 years after diagnosis. The patient continued on low dose prednisolone (5 mg per day). Ten years after diagnosis a biopsy was taken (*arrow*).

Abbreviations: DDD = dense deposit disease, MMF = mycophenolate mofetil, UPCR = Urine Protein-Creatinine Ratio (g/10mmol creatinine)

**Supplemental Figure S3A-F: kidney biopsy of patient 2 at diagnosis (A-C) and at follow-up (D-F).**

Legend: the first kidney biopsy contained 30 glomeruli per cross section. In light microscopy a membranoproliferative glomerulonephritis was seen, with nodular mesangial expansion, mesangial hypercellularity and hyperlobulation. There was mild endocapillary proliferation and in few glomeruli signs of recent extracapillary proliferation. Ribbon-like eosinophilic deposits were seen in the capillary walls (panel A, MS staining, magnification 40x). On IF there was intense staining for C3 (panel B) of the capillary wall and the mesangium, with moderate staining for IgM (++), C1q (+), Kappa (++) and Lambda (+). Staining for IgG and IgA was negative. EM (panel C) showed electron dense deposits in the lamina densa of the GBM and partial foot process effacement with moderate microvillus transformation of the podocytes.

The second biopsy, obtained 14 years after diagnosis, contained 35 glomeruli per cross section in LM. The biopsy disclosed a sclerosing glomerulopathy (30% globally sclerosed glomeruli, with 20% IFTA) with massive eosinophilic deposits in the mesangium and capillary walls (panel D). The nodular pattern with hyperlobulation was no longer observed. There was no mesangial, endocapillary, or extracapillary proliferation present (panel D, MS staining, magnification 40x). There was 20% IF/TA. On IF intense C3 staining was seen (+++), with negative staining for IgG, IgM, IgA, C1q, Kappa and Lambda. On EM massive intramembranous and tubular basement membrane dense deposits with extensive podocyte foot process effacement and increased microvillus transformation of the podocytes.

Abbreviations: EM = electron microscopy, IF = immunofluorescence, IF/TA = interstitial fibrosis and tubular atrophy, LM = light microscopy, MS = methenamine silver stain.

**Supplemental Figure S4: Clinical course of patient 2 after diagnosis of DDD**

Legend: at presentation with nephrotic syndrome the patient was started on oral prednisolone (60 mg per day) (*dark blue bar*). After clinical improvement the dose of prednisolone was decreased to 40 mg every other day. Two years after diagnosis complete remission was reached (UPCR 0.3 g/10mmol creatinine, serum albumin 36 g/L). Prednisolone was tapered and discontinued 5 years after diagnosis. Nine years after diagnosis recurrent nephrotic syndrome was diagnosed. The patient was treated with methylprednisolone pulses (3x 1000 mg i.v.) (*triangle*), followed by oral prednisolone (60 mg per day) and mycophenolate acid (2x 360 mg) (*light blue bar*). She again reached complete remission. Prednisolone was tapered and stopped and mycophenolate acid dose was reduced. Twelve years after diagnosis a 2^nd^ recurrence occurred. The dose of mycophenolate acid was increased (2x 540 mg) with no effect on proteinuria or serum albumin. Due to gastro-intestinal complaints the dose was recently reduced to 2x 360 mg. Fourteen years after diagnosis a biopsy was taken (*arrow*).

Abbreviations: DDD = dense deposit disease, MA = mycophenolate acid, UPCR = Urine Protein-Creatinine Ratio (g/10mmol creatinine)

Supplemental Table S1: Clinical parameters and histology of repeated kidney biopsies in patients treated with eculizumab.

| **Author** | **Age / diagnosis** | **Bx at baseline** | **sCr** (µmol/L) **before treatment with ECU** | **UPCR** (g/10mmol) **and sAlb** (g/L) **before treatment with ECU** | **Treatment** | **sCr at last FU on ECU** | **UPCR** (g/10mmol) **and sAlb** (g/L) **at last FU on ECU** | **Repeat Bx after treatment with ECU** | **Decrease in inflammation in repeated Bx** | **Comments** |
| --- | --- | --- | --- | --- | --- | --- | --- | --- | --- | --- |
| Bomback *et al*. 2012  Herlitz *et al.* 2012  [1, 2] | 22 yrs / DDD | Moderate mesangial proliferation, segmentally prominent endocapillary proliferation with leukocyte infiltration and focal membranoproliferative features. | 159 –177 | UPCR 0.37  sAlb 46 | Eculizumab | 115-124 | UPCR 0.66  sAlb 42 | Persistent mesangial proliferation but resolution of endocapillary proliferation and leukocyte infiltration. Unchanged degree of global glomerulosclerosis (20%), and tubulointerstitial scarring (15%). IF: Similar staining patterns and intensity for C3 and C5b-9. EM: mild decrease in mesangial and GBM deposits, increase in TBM deposits. | Yes |  |
| Bomback *et al*. 2012  Herlitz *et al.* 2012  [1, 2] | 42 yrs / DDD recurrence after Tx | Not described. | 133-150 | UPCR 12.0  sAlb 29 | Eculizumab | 150 | UPCR 0.23-2.15  sAlb 40 | Mild decrease in mesangial proliferation and resolution of neutrophil infiltration. IF: similar staining patterns and intensity for C3 and C5b-9. EM: decrease in mesangial dense deposits. | Yes |  |
| Bomback *et al*. 2012  Herlitz *et al.* 2012  [1, 2] | 25 yrs / C3GN | Diffuse MPGN, with prominent mesangial proliferation with membranoproliferative features, 50% global glomerulosclerosis and 40% tubulointerstitial scarring. | 141 | UPCR 2.57  sAlb 32 | Eculizumab, (added to MMF, pred) | 203  (after re-introduction of MMF/pred) | UPCR 1.68  sAlb 38  (after re-introduction of MMF/pred) | Active GN with persistent membranoproliferative changes and large subendothelial deposits. Increased chronicity, 85% sclerosis and 65% fibrosis.  IF: similar staining patterns and intensity for C3 and C5b-9. EM: increase in sub-endothelial deposits. | No | MMF/pred was stopped during eculizumab treatment, resulting in increase of UPCR (2.2), sCr (212) and decrease of sAlb (31). Repeat Bx was taken in this period. |
| Bomback *et al*. 2012  Herlitz *et al.* 2012  [1, 2] | 22 yrs / C3GN recurrence in Tx | Prominent mesangial and endocapillary proliferation with abundant leukocyte infiltration. | 150-168 | UPCR 5.04  sAlb 34 | Eculizumab (added to IS for the Tx, including prednisone) | 159 | UPCR 5.42  sAlb 31 | Decrease in endocapillary proliferation and leukocyte infiltration with a mild increase in chronic glomerular scarring and interstitial fibrosis. IF: similar staining patterns and intensity for C3 and C5b-9. EM: unchanged deposits. | Yes | After discontinuation of eculizumab AKI with in repeat biopsy marked increase in inflammation and development of crescents. |
| Bomback *et al*. 2012  Herlitz *et al.* 2012  [1, 2] | 20 yrs / G3GN recurrence in Tx | Mild mesangial proliferation with no endocapillary proliferation or exudative features. | 159 | UPCR 0.09  sAlb 43 | Eculizumab (added to MMF, tacro, pred) | 124 | UPCR 0.12  sAlb 50 | No significant change: mild mesangial proliferation with no endocapillary proliferation or exudative features. No change in IF and EM findings. | No | Prednisone was stopped during eculizumab therapy. |
| Vivarelli *et al.*  2012  [3] | 17 yrs / DDD | Dense deposits disease with focal sclerosis in 40% of the glomeruli. (Not described in detail) | 99 | 3.5-5.5 g per 24 hours  sAlb unknown | Eculizumab | Normal renal function | 0.96 g per 24 hours  sAlb 47 | Two repeat biopsies were performed at 6 and 18m after start of eculizumab: progressive reduction in mesangial proliferation and thickness of the glomerular capillary loops. Progression of glomerular sclerosis and tubular atrophy.  IF: reduction in C3 and C5b-9 deposits.  EM: dense deposits appeared to be reduced at 18m. | Yes | Serum levels of complement C3 remained low. |
| Gurkan *et al.*  2013  [4] | 21 yrs / C3GN recurrence after Tx | Mesangial and endocapillary proliferation with patchy scarring. IF: strong C3 staining along the glomerular capillary wall and in the mesangium. EM: scattered elongated deposits, predominantly subendothelial with endocapillary inflammation and diffuse effacement of the foot processes. | 133 | UPCR 2.26-3.39  sAlb unknown | Eculizumab | 106-124 | UPCR 1.47 (after max dose of ACEi/ARB)  sAlb unknown | Two repeat biopsies were performed at 6 and 12m after start of eculizumab: increased chronicity with 20% fibrosis, similar degrees of mesangial and endocapillary proliferation, slightly more glomeruli with MGPN features. | No | After initial decrease, proteinuria increased while on eculizumab therapy. |
| Garg *et al.*  2018  [5] | 51 yrs / C3G recurrent after Tx | Widespread acute tubular injury, mild mesangial expansion with capillary wall C3 deposition. EM sub-epithelial, sub-endothelial and mesangial deposits. | HD dependent | UPCR 0.15g/L  sAlb unknown | Eculizumab (added to pred, MMF, tacro) | 186 | UPRC 0.01 g/L  sAlb unknown | Less tubule-interstitial injury, glomerular findings were unchanged. | No |  |
| Oosterveld *et al.*  2015  [6] | 13 yrs / DDD | Diffuse global mesangiocapillary GN, mesangial proliferation 3 (0-3), endocapillary 2.3 (0-3), some interstitial inflammation. | 126 | UPCR 19.2  sAlb unknown | Eculizumab (added to pred) | 106 (estimated) | UPCR 0.1-0.28 (estimated)  sAlb unknown | Unchanged histology: persistent mesangiocapillary GN and active tubule-interstitial nephritis. | No |  |
| Le Quintrec *et al.*  2015  [7] | 63 yrs / C3G recurrence after Tx | Endocapillary proliferation +++, glomerular inflammatory cells ++, crescents 0/12, C3 staining +++, C5b-9 staining ++. | 194 | UPCR 1.6  sAlb 35 | Eculizumab | 80 | UPCR 0.9  sAlb 37 | Endocapillary proliferation -, glomerular inflammatory cells -, crescents 0/21, C3 staining neg, C5b-9 staining neg. | Yes | Presentation with pure endocapillary proliferation |
| Welte *et al.*  2018  [8] | Mid-teens / C3GN | Mesangial proliferation pos, leukocyte infiltration 1 (0-3), IFTA 10%, global glomerular sclerosis 4/24. IF: C3 +++. | 71 | UPCR 6.33  sAlb unknown | Eculizumab (combined with MMF) | NA | No response | Mesangial proliferation pos, leukocyte infiltration 0 (0-3), IFTA 10%, global glomerular sclerosis 16/19. IF: C3 +++. | Yes |  |
| Kojc *et al*.  2019  [9] | 14 yrs / C3GN | Severe glomerular inflammatory activity with nearly 70% crescents, 46% segmental glomerulosclerosis, 70% interstitial fibrosis. | Unknown | NS | Eculizumab | Improvement of renal function | Improvement in proteinuria | Significant decrease in glomerular inflammatory activity. In subsequent biopsies increasing chronicity. IF: no change in C3 staining and intensity. | Yes |  |
| Le Quintrec *et al.* 2018  [10] | 14 yrs, C3GN | Double contours +, inflammatory cells in glomeruli +, mesangial proliferation ++, cellular crescents 9%, glomerulosclerosis 0%, interstitial fibrosis <5%, TA <10%, IF: C3 +++. | 53 | UPCR 3.4  sAlb 27 | Eculizumab (added to pred) | 53 | UPCR 0.5  sAlb 35 | Double contours +, inflammatory cells in glomeruli +/-, mesangial proliferation +, cellular crescents 0%, glomerulosclerosis 0%, interstitial fibrosis <5%, TA <10%, IF: C3 +++. | Yes |  |
| Le Quintrec *et al.* 2018  [10] | 12 yrs, C3GN | Double contours +, inflammatory cells in glomeruli +, mesangial proliferation ++, cellular crescents 0%, glomerulosclerosis 0%, interstitial fibrosis <10%, TA <10%, IF: C3 +++. | 53 | UPCR 1.5  sAlb 34 | Eculizumab (added to pred) | 53 | UPCR 2.5  sAlb 28 | Double contours +, inflammatory cells in glomeruli 0, mesangial proliferation +, cellular crescents 0%, glomerulosclerosis 9%, interstitial fibrosis <10%, TA <10%, IF: C3 +++. | Yes |  |
| Le Quintrec *et al.* 2018  [10] | 17 yrs, C3GN | Double contours ++, inflammatory cells in glomeruli +, mesangial proliferation +, cellular crescents 0%, glomerulosclerosis 11%, interstitial fibrosis <10%, TA 15%, IF: C3 +++. | 44 | UPCR 6.9  sAlb 27 | Eculizumab | 53 | UPCR 9.5  sAlb 20 | Double contours ++, inflammatory cells in glomeruli +/-, mesangial proliferation 0, cellular crescents 0%, glomerulosclerosis 28%, interstitial fibrosis <10%, TA 15%, IF: C3 +++. | Yes |  |
| Le Quintrec *et al.* 2018  [10] | 9 yrs, C3GN | Double contours ++, inflammatory cells in glomeruli ++, mesangial proliferation +, cellular crescents 0%, glomerulosclerosis 0%, interstitial fibrosis <10%, TA <10%, IF: C3 +++. | 18 | UPRC 8.3  sAlb 24 | Eculizumab (added to pred) | 44 | UPCR 1.9  sAlb 25 | Double contours ++, inflammatory cells in glomeruli +, mesangial proliferation +, cellular crescents 0%, glomerulosclerosis 24%, interstitial fibrosis <10%, TA <10%, IF: C3 +++. | Yes |  |
| Le Quintrec *et al.* 2018  [10] | 65 yrs, C3GN | Double contours 0, inflammatory cells in glomeruli 0, mesangial proliferation 0, cellular crescents 60%, glomerulosclerosis 17%, interstitial fibrosis 50%, TA >50%, IF: C3 ++/+++. | 451 | UPCR 2.9  sAlb 24 | Eculizumab | 159 | UPCR 3.4  sAlb 31 | Double contours 0, inflammatory cells in glomeruli 0, mesangial proliferation 0, cellular crescents 0%, glomerulosclerosis 28%, interstitial fibrosis 50%, TA 50%, IF: C3 +. | Yes |  |
| Le Quintrec *et al.* 2018  [10] | 46 yrs, C3GN | Double contours ++, inflammatory cells in glomeruli +++, mesangial proliferation ++, cellular crescents 0%, glomerulosclerosis 8%, interstitial fibrosis 28%, TA 15%, IF: C3 ++. | 362 | UPCR 13.0  sAlb 22 | Eculizumab | 97 | UPCR 2.1  sAlb 40 | Double contours +, inflammatory cells in glomeruli 0, mesangial proliferation 0, cellular crescents 0%, glomerulosclerosis 0%, interstitial fibrosis 30%, TA 15-20%, IF: C3 ++. | Yes |  |
| Le Quintrec *et al.* 2018  [10] | 26 yrs, C3GN | Double contours ++, inflammatory cells in glomeruli +/++, mesangial proliferation 0, cellular crescents 60%, glomerulosclerosis 72%, interstitial fibrosis 25%, TA 25-30%, IF: C3 ++. | 530 | UPCR 13.6  sAlb 18 | Eculizumab | 194 | UPCR 0.8  sAlb 38 | Double contours +, inflammatory cells in glomeruli 0, mesangial proliferation 0, cellular crescents 0%, glomerulosclerosis 53%, interstitial fibrosis 20%, TA 20%, IF: C3 ++. | Yes |  |
| Le Quintrec *et al.* 2018  [10] | 24 yrs, C3G | Double contours +++, inflammatory cells in glomeruli 0, mesangial proliferation +, cellular crescents 0%, glomerulosclerosis 29%, interstitial fibrosis 30%, TA 20-25%, IF: C3 +++. | 71 | UPCR 7.0  sAlb 15 | Eculizumab | 71 | UPCR 1.6  sAlb 32 | Double contours +, inflammatory cells in glomeruli 0, mesangial proliferation ++/+++, cellular crescents 0%, glomerulosclerosis 12%, interstitial fibrosis <5%, TA <10%, IF: C3 +++. | No |  |
| Le Quintrec *et al.* 2018  [10] | 27 yrs, C3G | Double contours +++, inflammatory cells in glomeruli 0, mesangial proliferation ++, cellular crescents 0%, glomerulosclerosis 20%, interstitial fibrosis 50%, TA 50%, IF: C3 +++. | 159 | UPCR 11.1  sAlb 26 | Eculizumab | 150 | UPCR 6.9  sAlb 27 | Double contours +++, inflammatory cells in glomeruli 0, mesangial proliferation ++, cellular crescents 0%, glomerulosclerosis 13%, interstitial fibrosis 40%, TA 40%, IF: C3 +++. | No |  |
| Le Quintrec *et al.* 2018  [10] | 42 yrs, C3G | Double contours 0, inflammatory cells in glomeruli 0, mesangial proliferation +/-, cellular crescents 0%, glomerulosclerosis 11%, interstitial fibrosis <5%, TA <10%, IF: C3 ++. | 53 | UPCR 5.8  sAlb 22 | Eculizumab | 53 | >3 g per 24 hours  sAlb 25 | Double contours 0, inflammatory cells in glomeruli 0, mesangial proliferation 0, cellular crescents 0%, glomerulosclerosis 14%, interstitial fibrosis <5%, TA <10%, IF: C3 +/++. | No |  |
| Le Quintrec *et al.* 2018  [10] | 23 yrs, DDD | Double contours +++, inflammatory cells in glomeruli 0, mesangial proliferation +, cellular crescents 7%, ATN, glomerulosclerosis 26%, interstitial fibrosis 15%, TA 0%, IF: C3 +++. | 124 | UPCR 1.5  sAlb 21 | Eculizumab | 256 | UPCR 2.3  sAlb 34 | Double contours +++, inflammatory cells in glomeruli 0, mesangial proliferation 0, cellular crescents 0%, glomerulosclerosis 44%, interstitial fibrosis >50%, TA 0%, IF: C3 ++. | Yes |  |

Legend: in the above patients with C3G a repeat biopsy was performed *after* treatment with eculizumab. The repeat biopsy was compared to a baseline biopsy. The baseline biopsy is often the biopsy performed at presentation of disease. However, in some patients a biopsy was available shortly before the introduction of eculizumab. Clinical parameters (serum creatinine, serum albumin and proteinuria) are compared before and after treatment with eculizumab.

Abbreviations: ATN = acute tubular necrosis, Bx = kidney biopsy, C3G = C3 glomerulopathy, C3GN = C3 glomerulonephritis, DDD = dense deposit disease, ECU = eculizumab, GMB = glomerular basement membrane, NA = not applicable, neg = negative, NS= nephrotic syndrome, prot = proteinuria, sAlb = serum albumin, sCr = serum creatinine, TA = tubular atrophy, UPCR = urinary protein-creatinine ratio, yrs = years.

References of Supplemental Table S1:

1. Bomback AS, Smith RJ, Barile GR, Zhang Y, Heher EC, Herlitz L, Stokes MB, Markowitz GS, D'Agati VD, Canetta PA, Radhakrishnan J, Appel GB (2012) Eculizumab for dense deposit disease and C3 glomerulonephritis. Clinical journal of the American Society of Nephrology : CJASN 7:748-756.

2. Herlitz LC, Bomback AS, Markowitz GS, Stokes MB, Smith RN, Colvin RB, Appel GB, D'Agati VD (2012) Pathology after eculizumab in dense deposit disease and C3 GN. Journal of the American Society of Nephrology : JASN 23:1229-1237.

3. Vivarelli M, Pasini A, Emma F (2012) Eculizumab for the treatment of dense-deposit disease. The New England journal of medicine 366:1163-1165.

4. Gurkan S, Fyfe B, Weiss L, Xiao X, Zhang Y, Smith RJ (2013) Eculizumab and recurrent C3 glomerulonephritis. Pediatric nephrology (Berlin, Germany) 28:1975-1981.

5. Garg N, Zhang Y, Nicholson-Weller A, Khankin EV, Borsa NG, Meyer NC, McDermott S, Stillman IE, Rennke HG, Smith RJ, Pavlakis M (2018) C3 glomerulonephritis secondary to mutations in factors H and I: rapid recurrence in deceased donor kidney transplant effectively treated with eculizumab. Nephrology, dialysis, transplantation : official publication of the European Dialysis and Transplant Association - European Renal Association 33:2260-2265.

6. Oosterveld MJ, Garrelfs MR, Hoppe B, Florquin S, Roelofs JJ, van den Heuvel LP, Amann K, Davin JC, Bouts AH, Schriemer PJ, Groothoff JW (2015) Eculizumab in Pediatric Dense Deposit Disease. Clinical journal of the American Society of Nephrology : CJASN 10:1773-1782.

7. Le Quintrec M, Lionet A, Kandel C, Bourdon F, Gnemmi V, Colombat M, Goujon JM, Fremeaux-Bacchi V, Fakhouri F (2015) Eculizumab for treatment of rapidly progressive C3 glomerulopathy. American journal of kidney diseases : the official journal of the National Kidney Foundation 65:484-489.

8. Welte T, Arnold F, Kappes J, Seidl M, Haffner K, Bergmann C, Walz G, Neumann-Haefelin E (2018) Treating C3 glomerulopathy with eculizumab. BMC Nephrol 19:7.

9. Kojc N, Bahovec A, Levart TK (2019) C3 glomerulopathy in children: Is there still a place for anti-cellular immunosuppression? Nephrology (Carlton) 24:188-194.

10. Le Quintrec M, Lapeyraque AL, Lionet A, Sellier-Leclerc AL, Delmas Y, Baudouin V, Daugas E, Decramer S, Tricot L, Cailliez M, Dubot P, Servais A, Mourey-Epron C, Pourcine F, Loirat C, Fremeaux-Bacchi V, Fakhouri F (2018) Patterns of Clinical Response to Eculizumab in Patients With C3 Glomerulopathy. American journal of kidney diseases : the official journal of the National Kidney Foundation 72:84-92.
