# Supplementary figures and images for: Treatment-resistant nephrotic syndrome in dense deposit disease: complement-mediated glomerular capillary wall injury?
Source: Pediatr Nephrol. 2020 May 23;35(9):1791–5. doi: 10.1007/s00467-020-04600-9 (PMC7384995; doi:10.1007/s00467-020-04600-9)

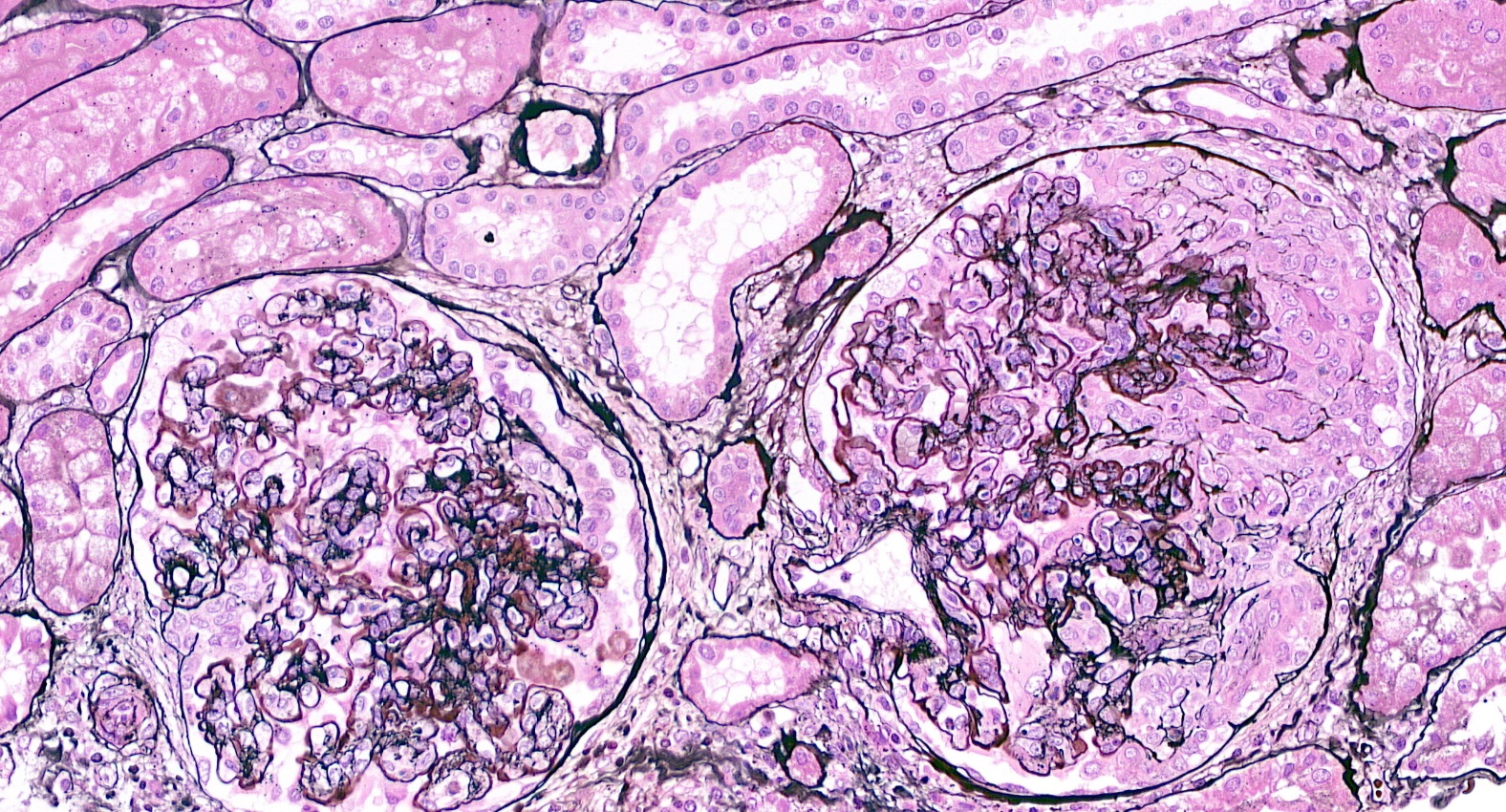

Supplement: Supplementary file 2 — (JPG 841 kb) [file 467_2020_4600_MOESM2_ESM.jpg]

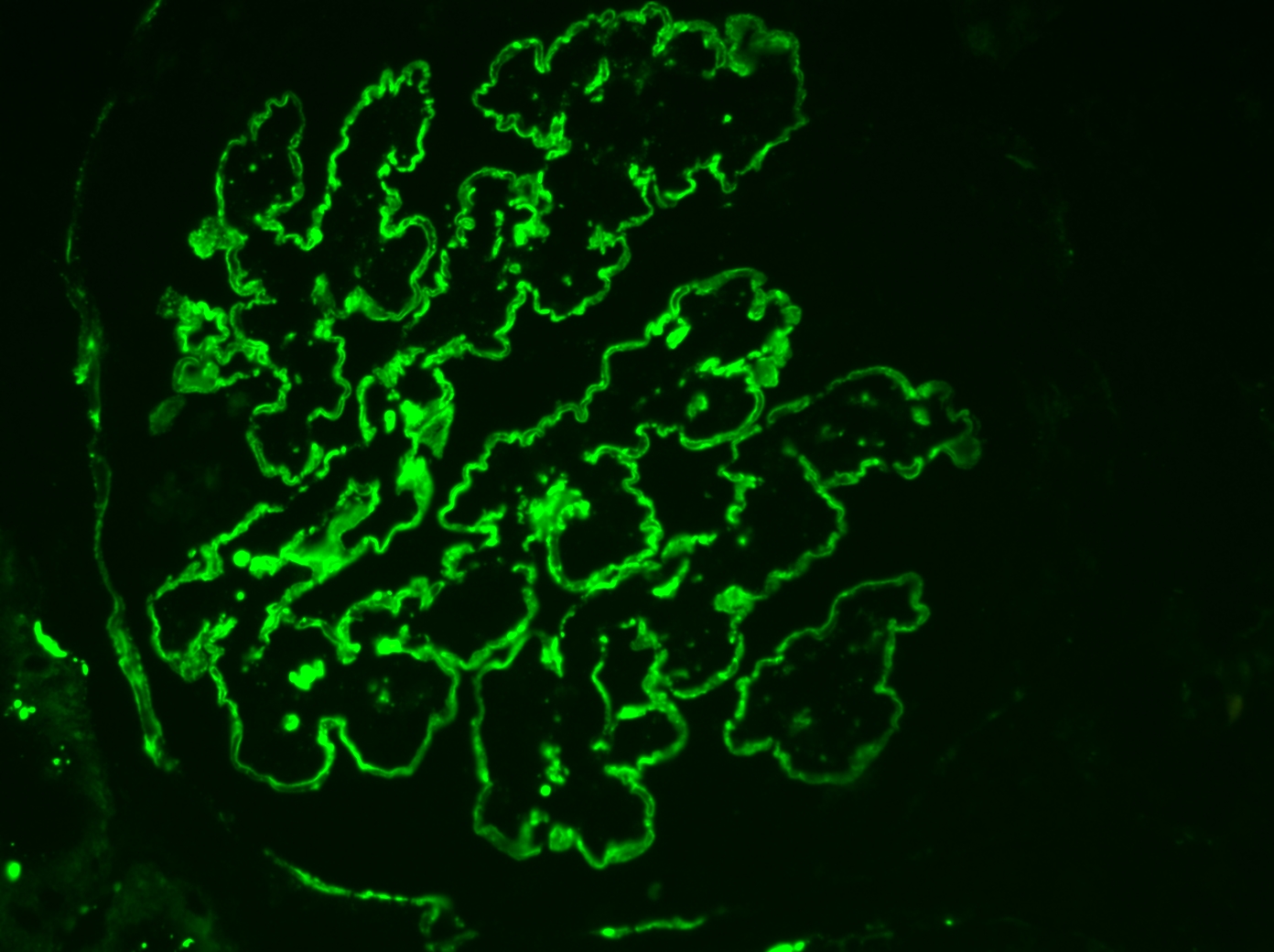

Supplement: Supplementary file 3 — (JPG 565 kb) [file 467_2020_4600_MOESM3_ESM.jpg]

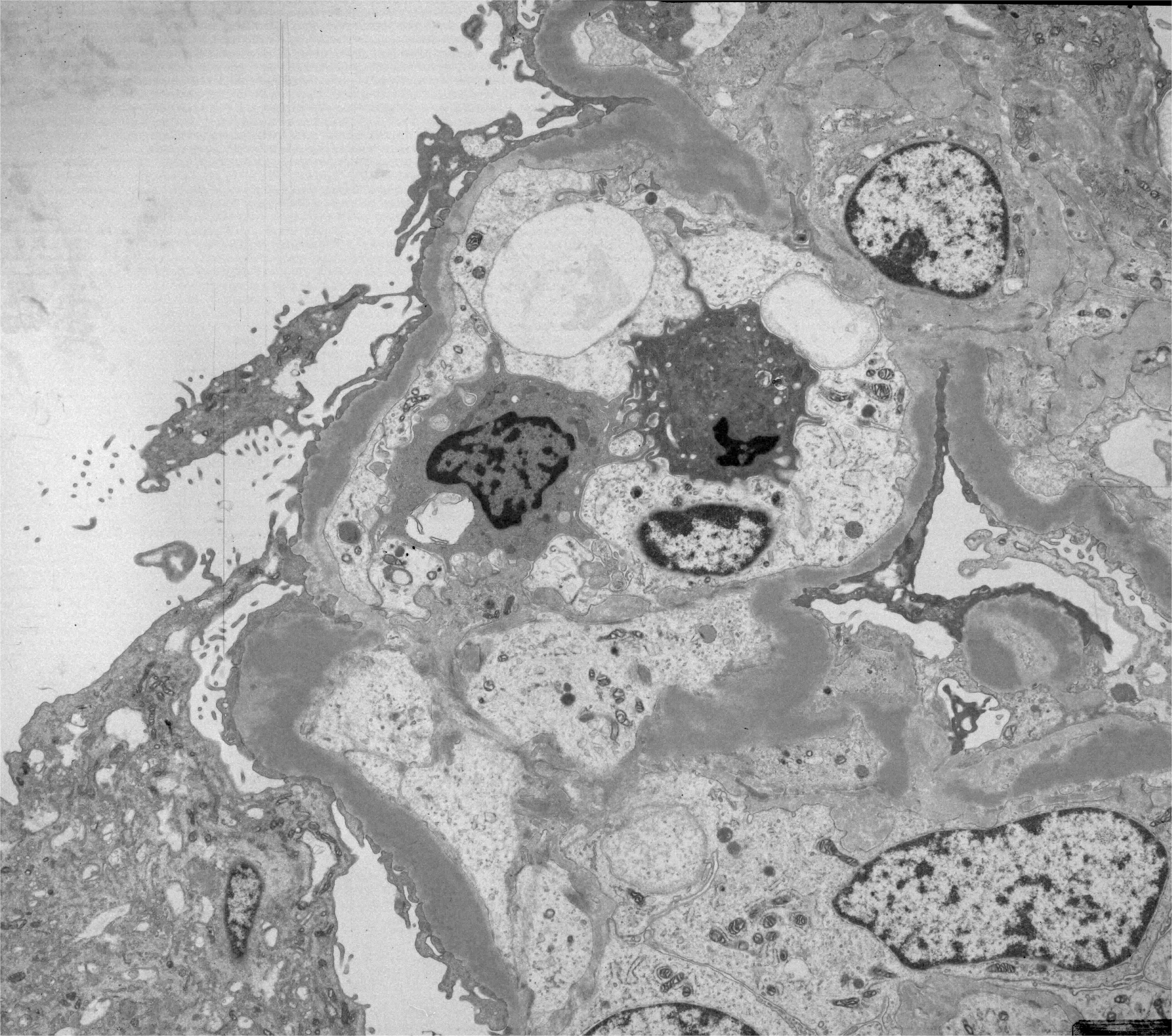

Supplement: Supplementary file 4 — (JPG 2617 kb) [file 467_2020_4600_MOESM4_ESM.jpg]

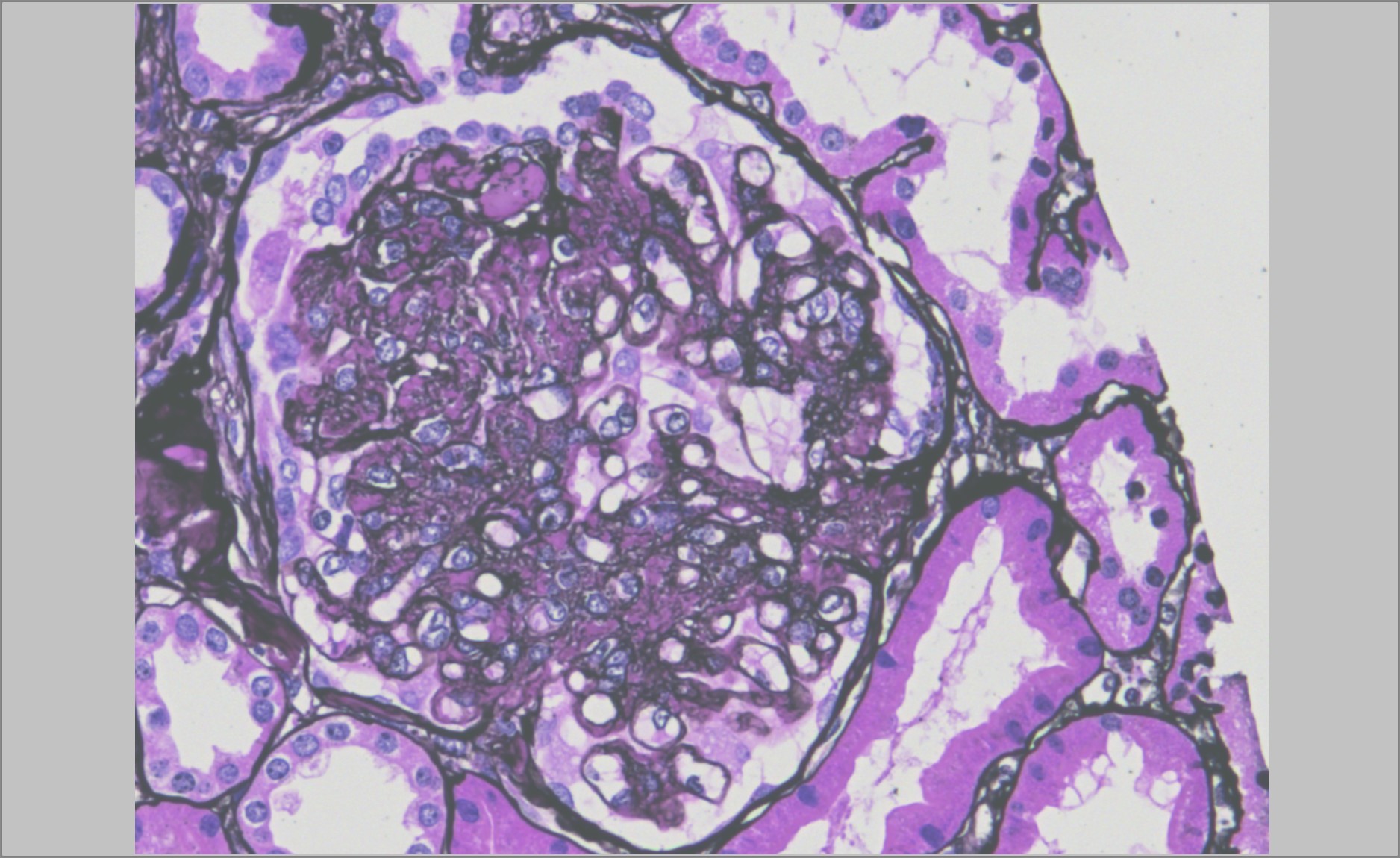

Supplement: Supplementary file 5 — (JPG 365 kb) [file 467_2020_4600_MOESM5_ESM.jpg]

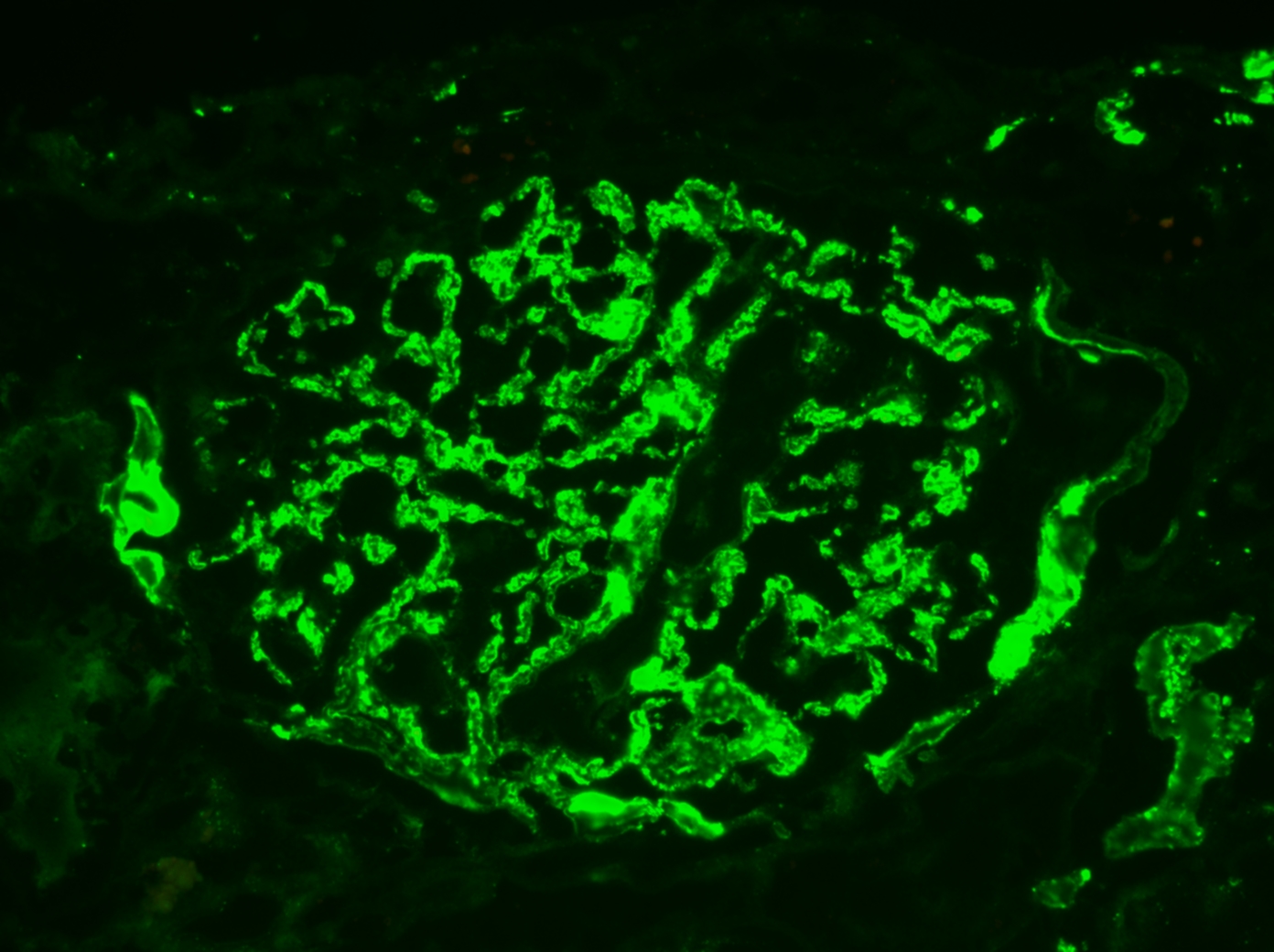

Supplement: Supplementary file 6 — (JPG 662 kb) [file 467_2020_4600_MOESM6_ESM.jpg]

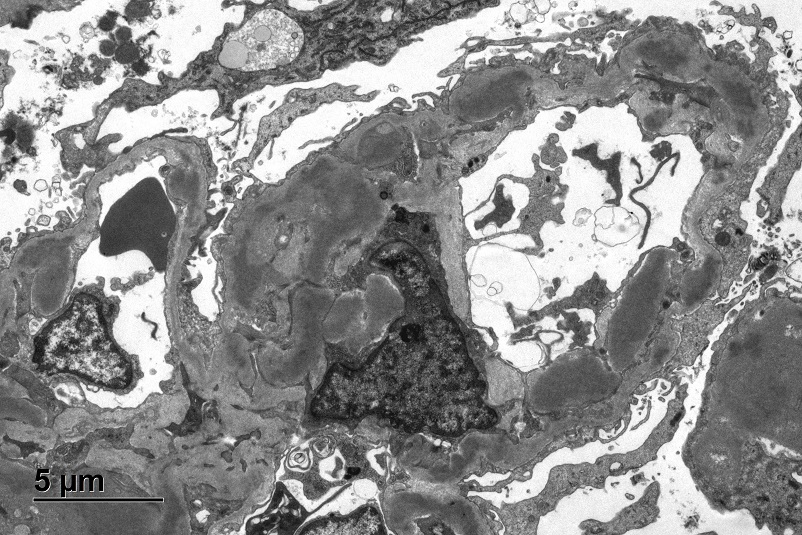

Supplement: Supplementary file 7 — (JPG 234 kb) [file 467_2020_4600_MOESM7_ESM.jpg]

## Slide 1
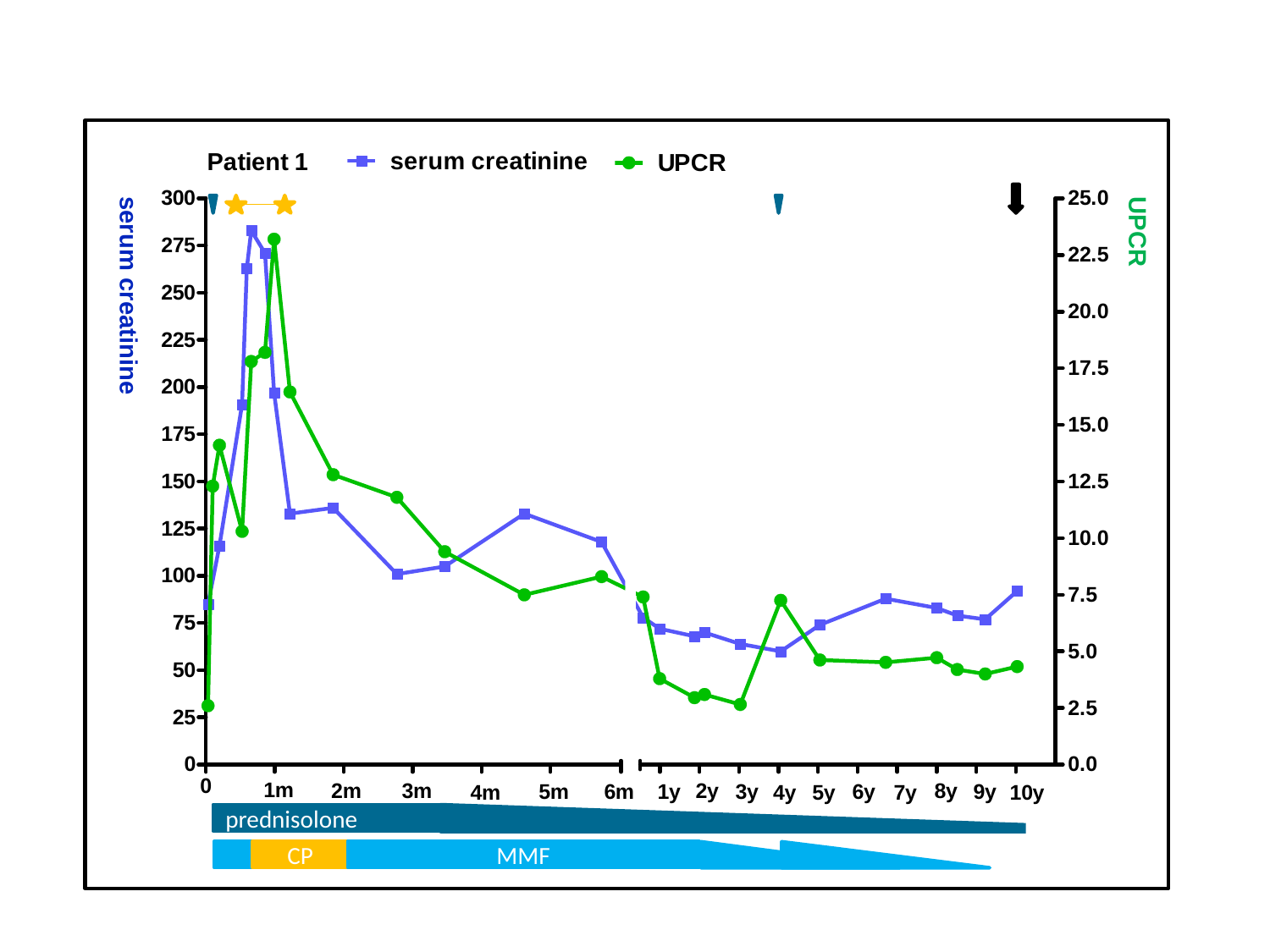

UPCR
serum creatinine
prednisolone
MMF
CP

Supplement: Supplementary file 8 — (PPTX 90 kb) [file 467_2020_4600_MOESM8_ESM.pptx]

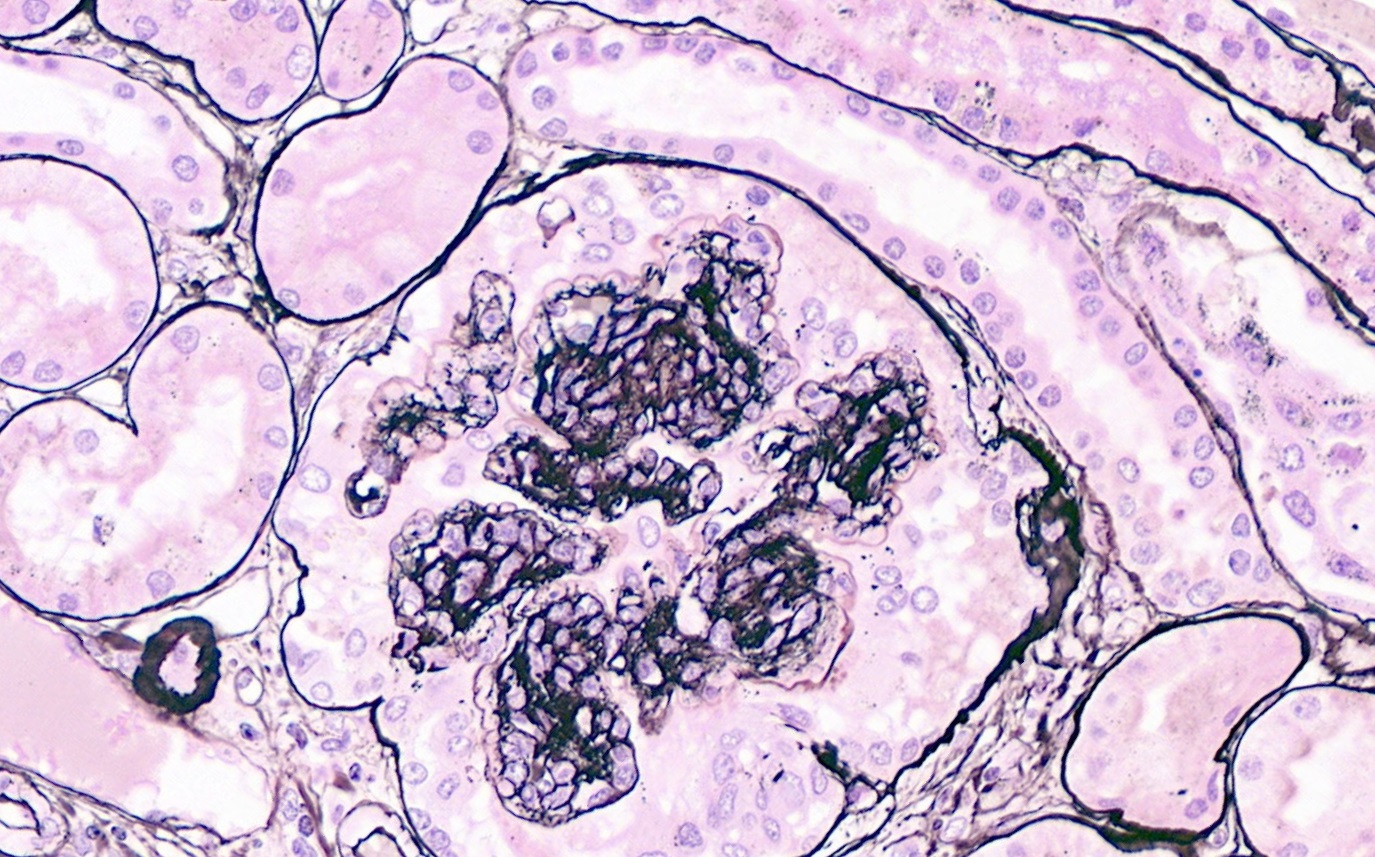

Supplement: Supplementary file 9 — (JPG 472 kb) [file 467_2020_4600_MOESM9_ESM.jpg]

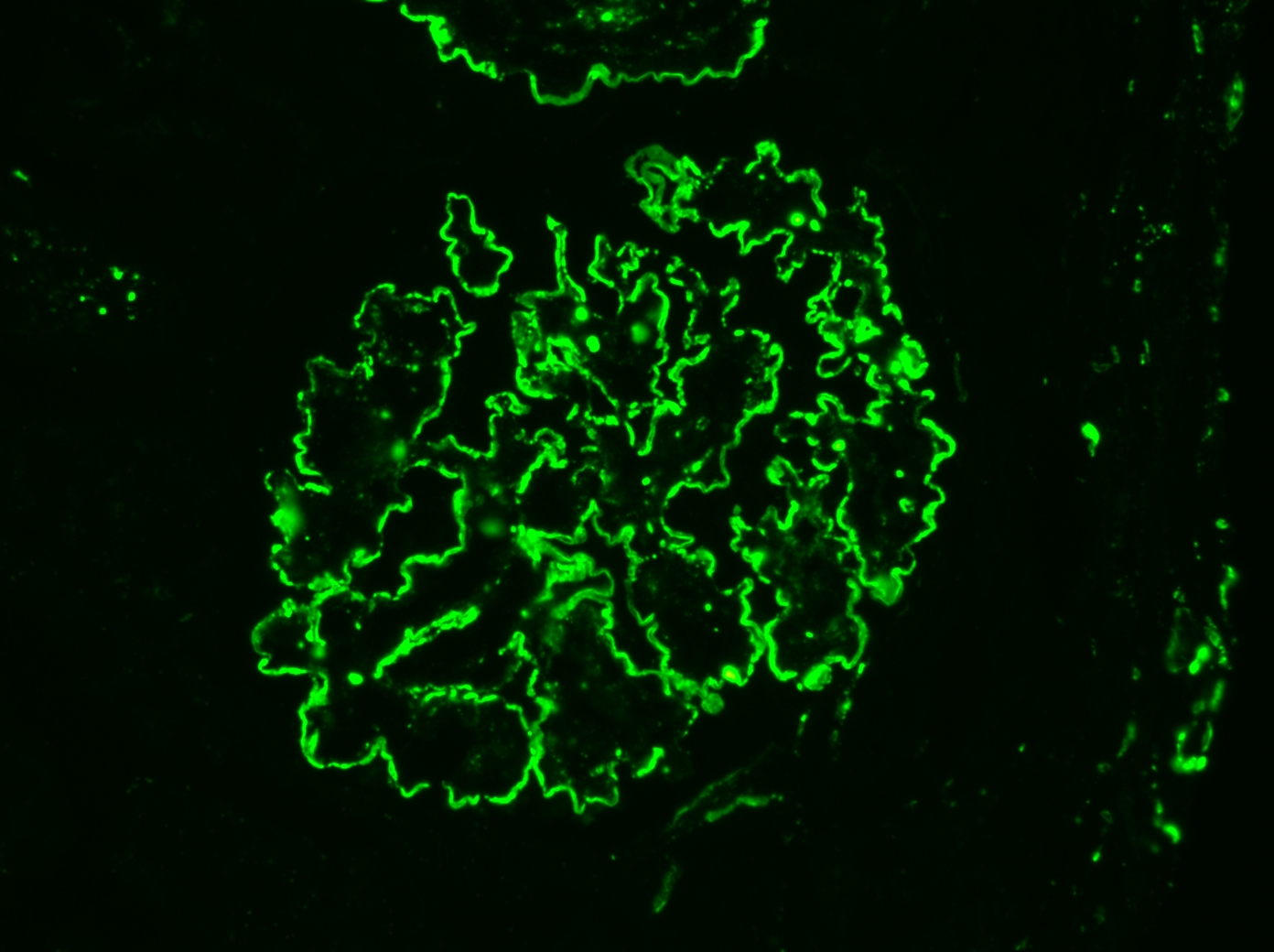

Supplement: Supplementary file 10 — (JPG 548 kb) [file 467_2020_4600_MOESM10_ESM.jpg]

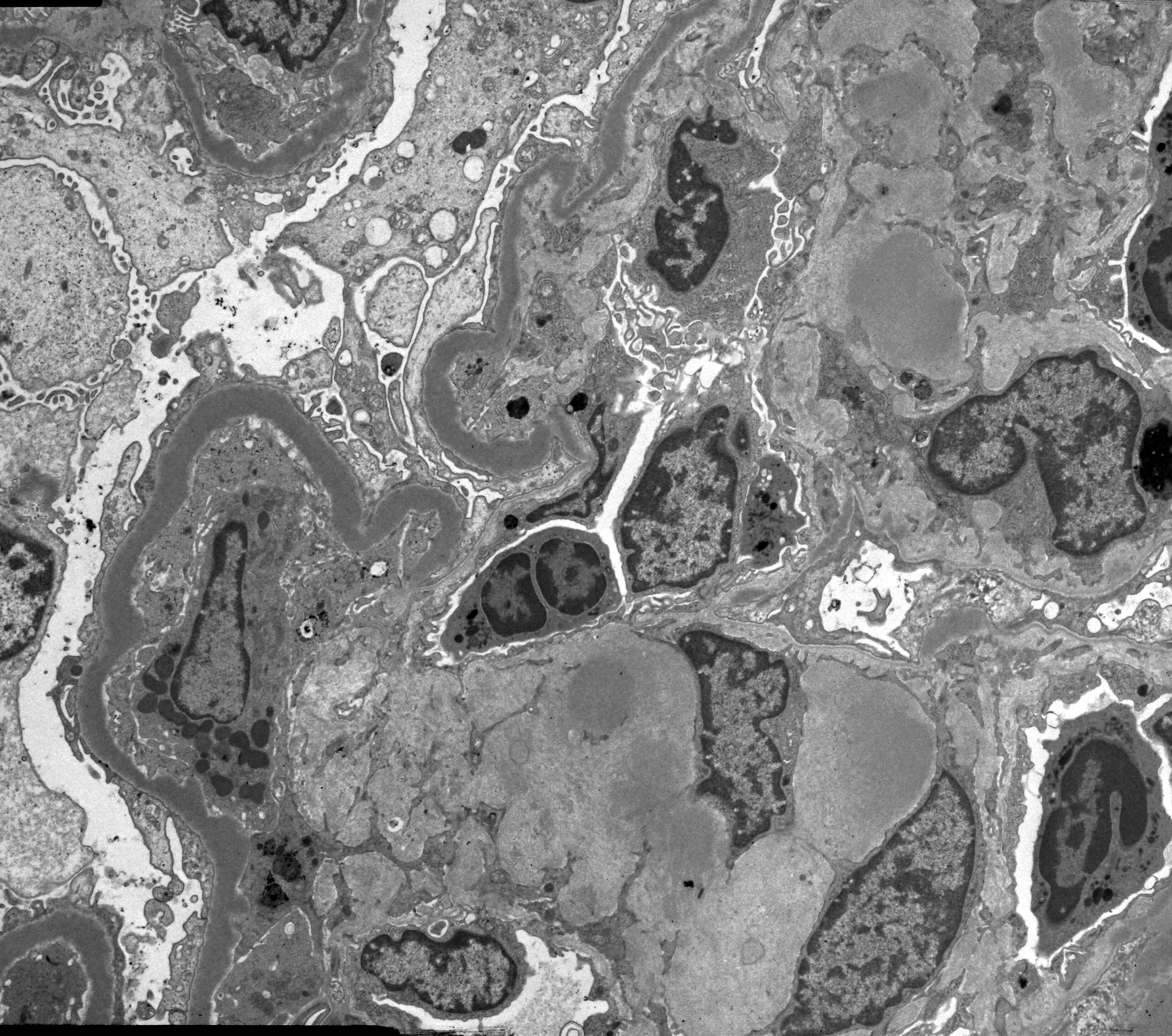

Supplement: Supplementary file 11 — (JPG 2588 kb) [file 467_2020_4600_MOESM11_ESM.jpg]

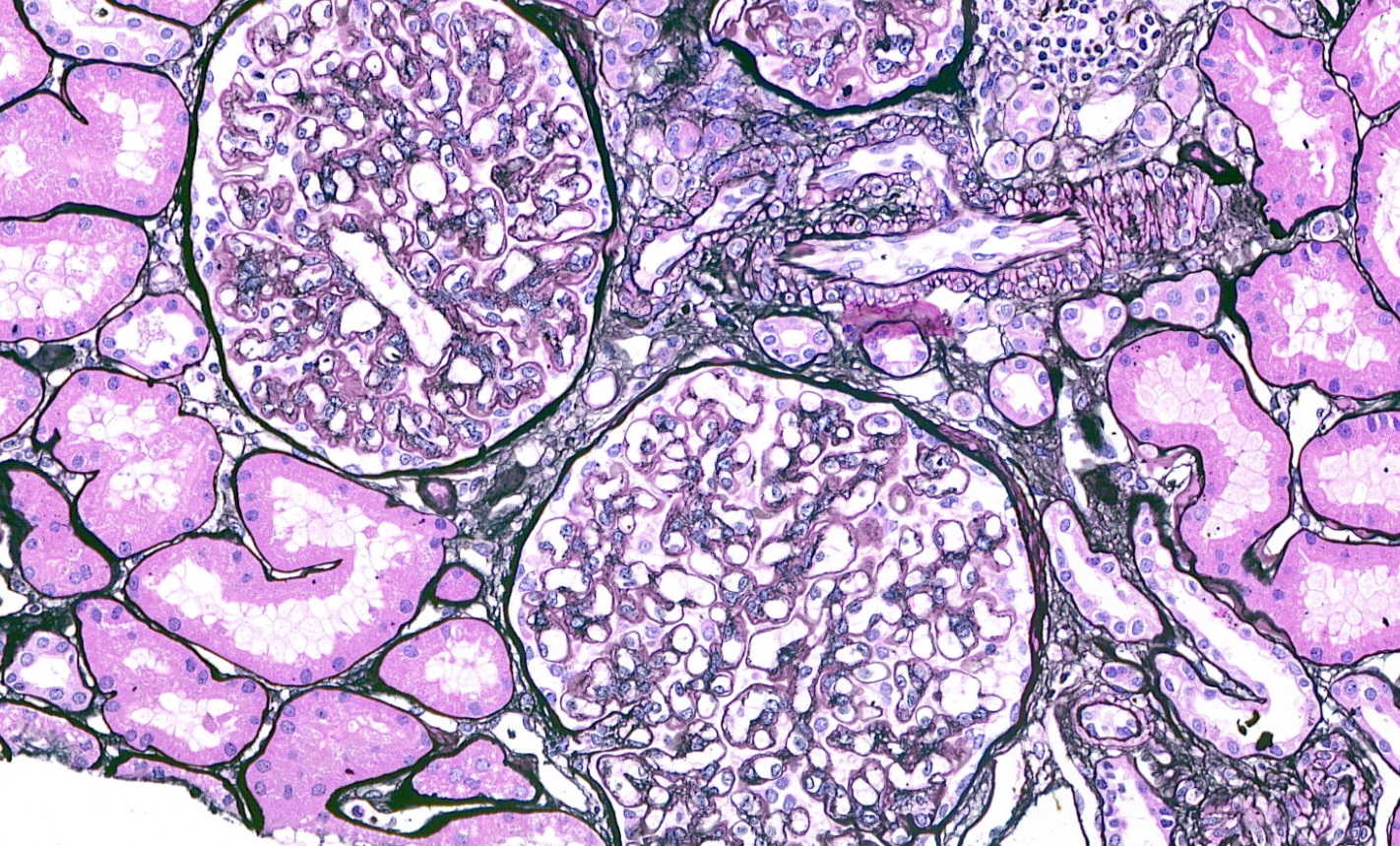

Supplement: Supplementary file 12 — (JPG 721 kb) [file 467_2020_4600_MOESM12_ESM.jpg]

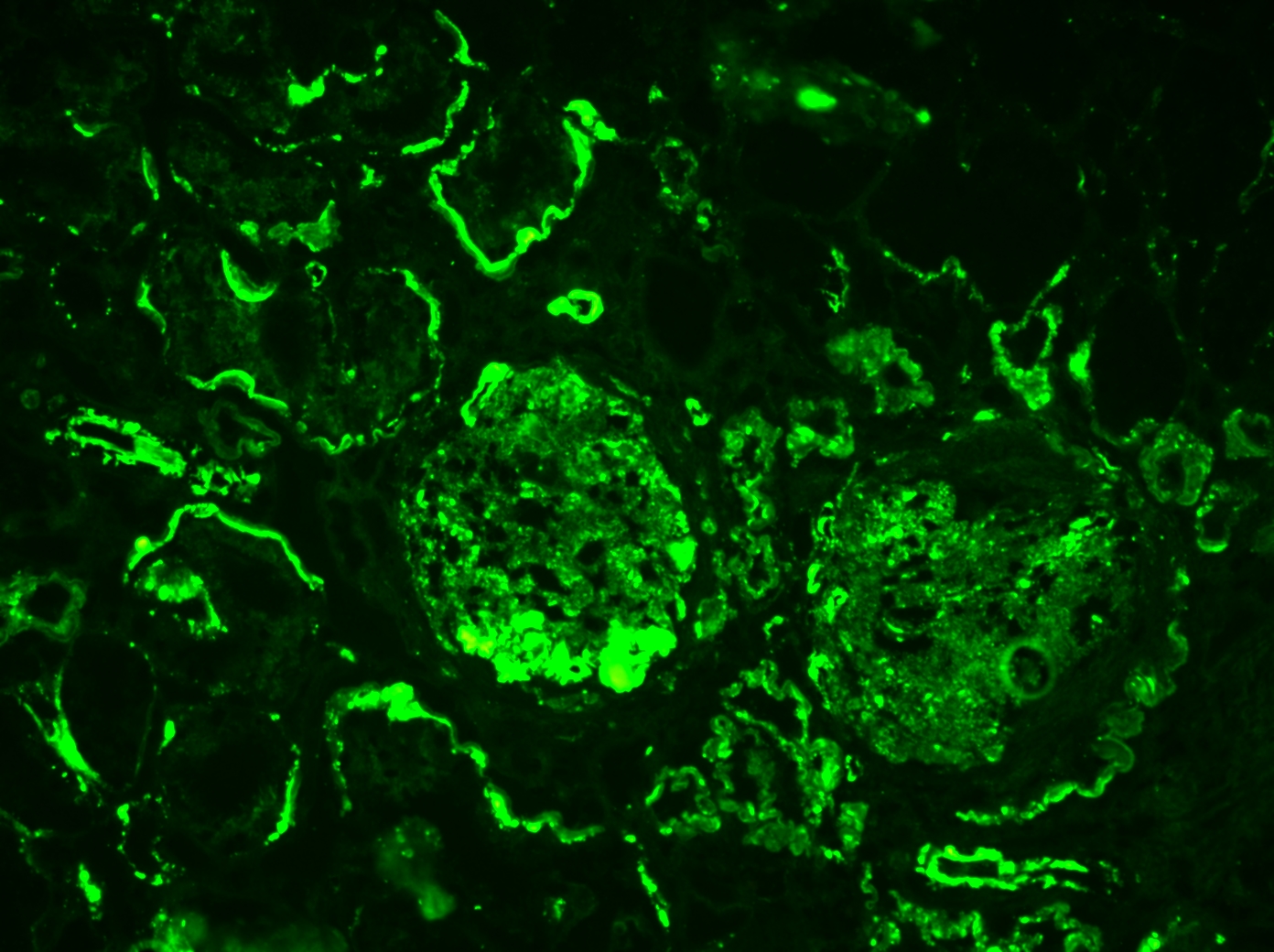

Supplement: Supplementary file 13 — (JPG 802 kb) [file 467_2020_4600_MOESM13_ESM.jpg]

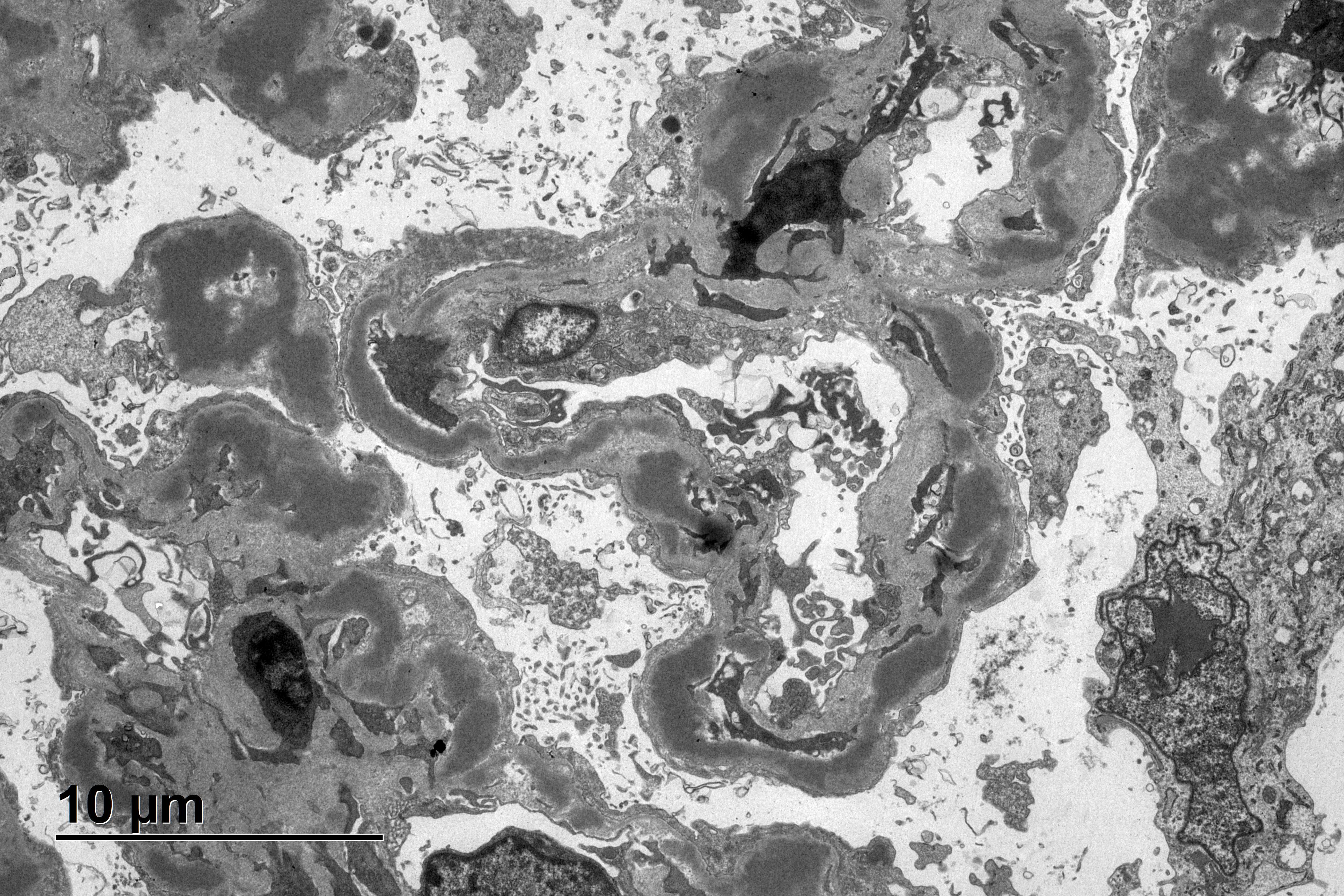

Supplement: Supplementary file 14 — (JPG 1760 kb) [file 467_2020_4600_MOESM14_ESM.jpg]

## Slide 1
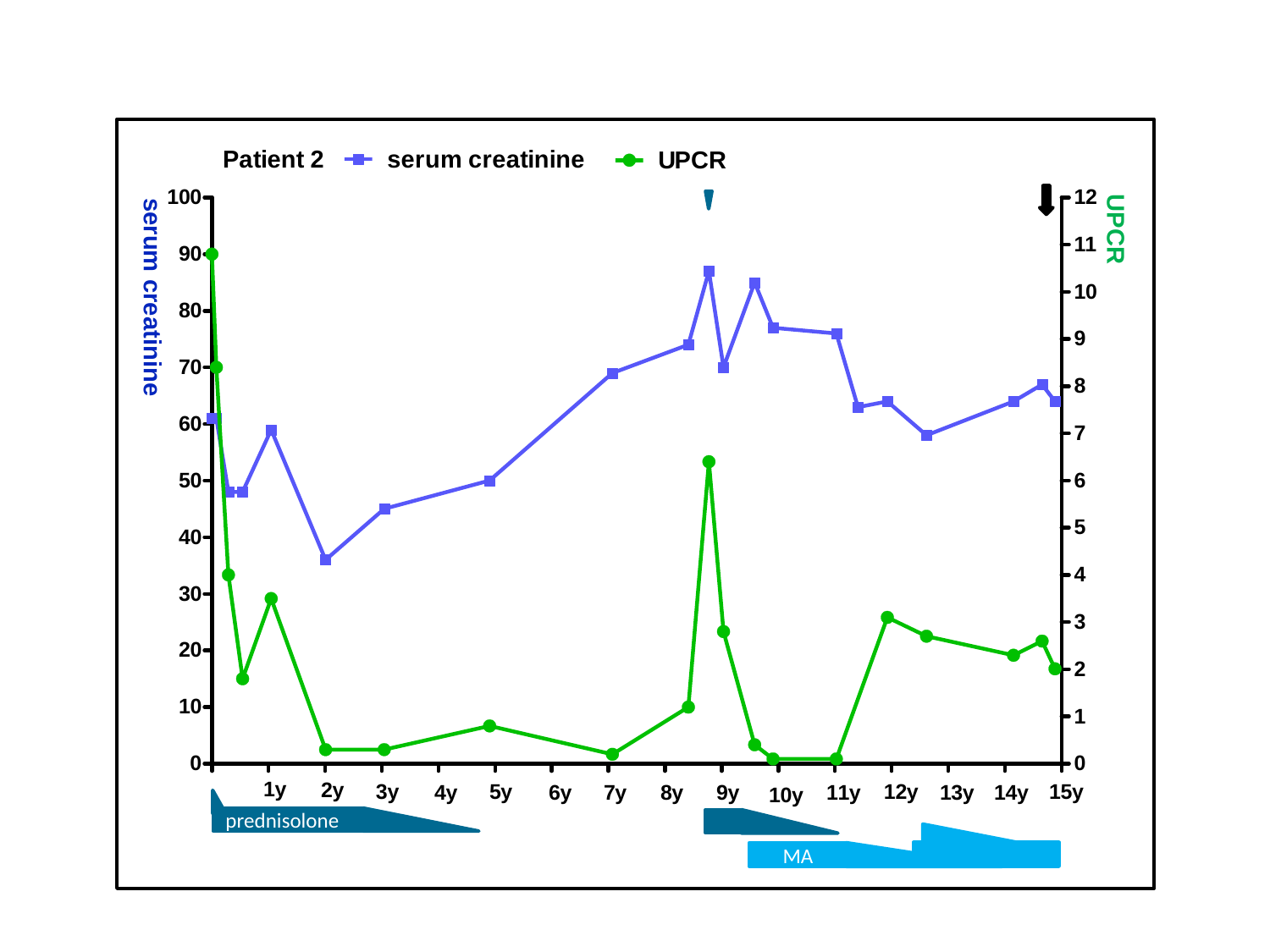

UPCR
serum creatinine
prednisolone
MA

Supplement: Supplementary file 15 — (PPTX 87 kb) [file 467_2020_4600_MOESM15_ESM.pptx]
